# Peptide receptor radionuclide therapy (PRRT) with [^177^Lu-DOTA^0^,Tyr^3^]octreotate in combination with RAD001 treatment: further investigations on tumor metastasis and response in the rat pancreatic CA20948 tumor model

**DOI:** 10.1186/s13550-014-0021-y

**Published:** 2014-05-30

**Authors:** Sander M Bison, Stefan E Pool, Stuart J Koelewijn, Linda M van der Graaf, Harald C Groen, Marleen Melis, Marion de Jong

**Affiliations:** 1 Department of Nuclear Medicine, Erasmus MC, Postbus 2040, Rotterdam 3000, CA, the Netherlands; 2 Department of Radiology, Erasmus MC, Rotterdam 3000, CA, the Netherlands; 3 Department of Medical Informatics, Erasmus MC, Rotterdam 3000, CA, the Netherlands

**Keywords:** RAD001, Everolimus, Affinitor, Metastasis, 177Lu-DOTATATE

## Abstract

**Background:**

Previously, we reported on the unexpected development of distant metastases in the subcutaneous rat pancreas CA20948 tumor model after 4.5 weeks of treatment with RAD001-only or in combination with [^177^Lu-DOTA^0^,Tyr^3^]octreotate (^177^Lu-DOTATATE) (Cancer Res. 73:12-8, 2013). Moreover, the combination therapy was less effective compared to ^177^Lu-DOTATATE-only. In the current study, we address the following questions: (1) Why was the combination therapy less effective? Is ^177^Lu-DOTATATE tumor uptake affected by pretreatment with RAD001? (2) Could sudden cessation of RAD001 therapy cause the development of distant metastases? (3) Is ^177^Lu-DOTATATE an effective treatment option for these metastases?

**Methods:**

Lewis rats (HanHsd or SsNHsd substrain with a slight difference in immune response) bearing subcutaneous CA20948 tumors were treated with either 125 or 275 MBq ^177^Lu-DOTATATE, RAD001, or their combination. RAD001 was given twice a week for 4.5 or 12 weeks, whereas ^177^Lu-DOTATATE was given as a single injection. When combined, RAD001 was started either 3 days prior to or 3 days post administration of ^177^Lu-DOTATATE. SPECT/CT was performed to quantify ^177^Lu-DOTATATE tumor uptake. Where indicated, primary tumors were surgically removed when tumor size is >6,000 mm^3^ to enable monitoring for possible metastasis. If metastases were suspected, an ^111^In-DTPA-octreotide SPECT/CT scan was performed. Seven rats with metastases were treated with 400 MBq ^177^Lu-DOTATATE.

**Results:**

Lu-DOTATATE tumor uptake was not significantly affected by RAD001 pretreatment. The occurrence of metastases after RAD001 treatment was not dose dependent in the dose range tested, nor was it related to the duration of RAD001 treatment. In the experiment in which the LEW/SsNsd substrain was used, only 12.5% of RAD001-treated rats showed complete response (CR), compared to 50% tumor regression in the control group. Re-treatment with a high dose of ^177^Lu-DOTATATE resulted in CR in only two out of seven animals.

**Conclusion:**

Less effective anti-tumor effects after the combination of RAD001 + ^177^Lu-DOTATATE could not be explained by reduced ^177^Lu-DOTATATE tumor uptake after RAD001. Our current data support RAD001-induced immune suppression as the reason for this observation. No evidence was found that cessation of RAD001 treatment caused development of metastases. Metastases appeared to be less sensitive to ^177^Lu-DOTATATE treatment than primary tumors.

## Background

Neuroendocrine tumors (NETs) consist of a heterogeneous group of neoplasms originating from cells characterized by the synthesis and release of amines/peptides [1]. Since 1973, the incidence of NETs has been increasing, in which genetic factors might play a role [2] and in addition improved diagnostics contributed to a higher registered incidence of NETs [1]. Because NETs are slowly proliferating tumors, the prevalence of NETs is much higher than the incidence, resulting in a relatively high percentage of NET patients in the population of cancer patients [3]. In >50% of the patients, NETs are diagnosed at a relatively late stage, often with metastatic spread [2], which leaves little chance for curative surgery. As a consequence of the slow proliferation rate, most NETs are relatively resistant to chemotherapeutics.

Most NETs are characterized by overexpression of somatostatin receptors, mainly subtype 2 (sst_2_). Targeting these receptors by administration of somatostatin analogs radiolabeled with, e.g., beta particle-emitting radionuclides, such as ^177^Lu or ^90^Y, allows peptide receptor radionuclide therapy (PRRT) of NET patients. This therapeutic approach is being performed since more than 10 years and has proven to be an effective treatment option in patients with inoperable disease. Therapeutic responses result in a significantly longer overall survival time compared to other treatments such as chemotherapy or external beam radiation therapy [4-6]. PRRT also improves patient's self-assessed quality of life [7]. Although PRRT is a successful therapy, complete remissions (CR) in patients with metastasized disease are still rare, so there is an urgent need for improvement.

The combination of PRRT with the mammalian target of rapamycin (mTOR) inhibitor everolimus or RAD001 could be promising in this respect. Everolimus (RAD001) has recently received FDA approval for the treatment of pancreatic NETs. RAD001 has been reported to show anti-tumor and anti-angiogenic activity both *in vitro* as well as *in vivo*, since both tumor proliferation and tumor angiogenesis are regulated by mTOR [8]. The clinical RADIANT III trial, a randomized, double-blind, placebo-controlled, multicenter phase III trial in pancreatic neuroendocrine tumor (PNET) patients, showed a median progression-free survival of 11 months after daily administration of 10 mg RAD001 plus best supportive care versus 4.6 months in the placebo group [2]. Since it was shown that RAD001 may act as radiosensitizer in various tumor models [9], RAD001 and PRRT could have a synergistic effect. Antitumor efficacies of RAD001 treatment schedules in the CA20948 tumor model have been reported before by Boulay et al. [10]. In this study, comparable anti-tumor effects were shown for twice weekly and daily RAD001 administration.

We have performed a combination study of the two treatments in the sst_2_-expressing CA20948 tumor-bearing rat model on which we recently reported the first data [11]. In this study, we compared either ^177^Lu-DOTATATE, RAD001, or their combination for treatment of tumor-bearing rats. RAD001 was administered orally twice a week for 4.5 weeks, and a suboptimal dose of ^177^Lu-DOTATATE (leaving room for additional effect of RAD001) was given once. Unexpectedly, we observed that the majority (77%) of rats treated with RAD001 (single treatment or combined with PRRT) developed metastases. We have used this subcutaneous CA20948 tumor model in many PRRT studies for more than 10 years, and metastases never occurred before. We have hypothesized that a rebound effect after stopping the RAD001 treatment after 4.5 weeks initiated a metastasizing process. Furthermore, we observed that rats treated with the combination of RAD001 and ^177^Lu-DOTATATE showed less impressive anti-tumor effects compared to those treated with ^177^Lu-DOTATATE-only.

In the current studies, we performed additional *in vivo* experiments in the same rat model and obtained additional results from the previous studies. To find an explanation for the lower therapeutic effect of the combination vs. single ^177^Lu-DOTATATE therapy, ^177^Lu-DOTATATE tumor uptake was quantified in tumors with and without RAD001 pretreatment. Moreover, in LEW/SsNsd rats, we studied the effects of longer, i.e., 12 instead of 4.5 weeks, RAD001 treatment on the potential induction of metastasis.

## Methods

### Tumor cell lines

The rat sst_2_-expressing pancreatic tumor CA20948 cell line [12] was cultured in Dulbecco's modified Eagle's medium (DMEM, Gibco, Invitrogen Corp., Breda, the Netherlands) supplemented with 10% heat-inactivated fetal bovine serum.

### Animals and tumor model

The animal ethics committee of our institution has approved all experiments. Male Lewis rats (250 to 300 g) were obtained from Harlan (Heerlen, the Netherlands). We used LEW/HanHsd Lewis rats, and where indicated, we also included rats from the LEW/SsNHsd substrain. In the LEW/SsNHsd substrain, the immune system shows an enhanced CD4+ and CD8+ T cell (auto)-immune response [13-17]. This autoimmunity is linked to an increased tumor immunity [18,19]. One week after arrival, rats were inoculated subcutaneously with 10^7^ CA20948 cells in 0.5 ml HBSS. For all experiments, animals were randomized into matching treatment groups with regard to tumor size at the start of treatment. A person blinded for the treatment measured tumor size using a calliper and weighed the rats three times a week. Tumor volume was calculated using the formula: 0.4 × length × weight × height. Tumor response was defined as follows: partial response (PR) as >50% reduction of tumor volume and complete response (CR) as 100% reduction of tumor volume. Tumors were allowed to develop until a maximum of 4 to 6 cm^3^ and were surgically removed where indicated. Rats were euthanized when >10% loss of body weight (BW) was registered.

### Anesthesia

2.5% Isoflurane/O_2_ gas anesthesia was used at 0.5 ml/min during tumor cell inoculation, administration of ^177^Lu-DOTATATE, scanning, or surgery.

### Surgical procedure to remove the primary tumor

When primary tumors were >4 to 6 cm^3^, surgical tumor resection was performed where indicated. During surgery, a heating pat was used to maintain body temperature. Routine shaving and disinfecting of the skin was performed. The tumor including the tumor capsule was carefully dissected from the surrounding tissue. After tumor resection, the skin was closed using individual sutures (vicryl 3/0).

### RAD001

RAD001 and its placebo, kindly provided by Novartis Pharmaceuticals, Basel, Switzerland, were used for study 1 and prepared according to the manufacturer's protocol. For the next experiments, RAD001 powder from LC laboratories, Woburn, USA, was dissolved in 2 ml ethanol and diluted with 5% glucose solution in water to obtain 3 or 6 mg/ml. RAD001 was administered orally by gavage in a volume of 0.2 ml; thus, 0.8 or 1.6 mg/rat or 2.5/5 mg/kg BW was administered depending on treatment group. For each administration, RAD001 was prepared freshly from powder.

### Radionuclides and peptides

DOTA^0^,Tyr^3^-octreotate was obtained from Mallinckrodt, St. Louis, MO, USA, and ^177^LuCl_3_ was obtained from IDB, Baarle-Nassau, the Netherlands. ^177^Lu-DOTATATE was prepared as described before [20], with a specific activity of 100 MBq/2.75 μg peptide, and injected iv via the tail vein under anesthesia. Labeling of ^111^In-DTPA-octreotide (OctreoScan, Covidien, Petten, the Netherlands) in a specific activity of 30 MBq/0.5 μg DTPA-octreotide was performed as described previously [21].

### SPECT/CT scanning

Forty-eight hours after injection of ^177^Lu-DOTATATE, helical single-photon emission computed tomography/computed tomography (SPECT/CT) scanning of the tumor region was performed with the four-headed NanoSPECT/CT system (BioScan, Washington DC, USA). Multi-pinhole rat collimators with nine pinholes (diameter 2.5 mm) per head were used: 24 projections, 90 s per projection, were applied. SPECT scans were reconstructed iteratively on a 256 × 256 matrix, using HiSPECT NG software (Scivis GmbH, Göttingen Germany) and ordered subset expectation maximization (OSEM). The total amount of radioactivity (MBq) in the tumor was quantified by drawing a sufficiently large volume of interest (VOI) around the tumor using InVivoScope software (IVS, Bioscan, Washington DC, USA). To achieve accurate quantification, the camera was calibrated by scanning a 20-ml polypropylene tube rat phantom filled with a known amount of ^177^Lu activity. The *in vivo* tumor volume was assessed by setting the lower threshold to 90% of the maximum voxel intensity of the tumor using the IRW program (Siemens, Erlangen, Germany). Before euthanasia, a whole-body SPECT/CT scan of rats was acquired 24 h after intravenous injection of ^111^In-DTPA-octreotide (50 MBq ^111^In/0.5 μg DTPA-octreotide) to detect CA20948 metastases. During scanning, the rat body temperature was maintained using a heated bed.

### *In vitro* autoradiography

Autoradiography was performed on primary tumors as well as metastases. Frozen sections of 10 μm (Cryo-Star HM 560 M; Microm, Walldorf, Germany) were mounted on Superfrost plus slides (Menzel, Braunschweig, Germany) and incubated with 10^−10^ M ^111^In-DTPA-octreotide with and without an excess (10^−6^ M) of unlabeled octreotide. Adjacent sections were used for hematoxylin/eosin staining. Tumor sections were exposed to SR phosphor imaging screens (Packard Instruments Co., Meriden, USA) in X-ray cassettes. After a 48-h exposure, screens were read by a Cyclone phosphor imager and analyzed using OptiQuant 03.00 (PerkinElmer, Groningen, the Netherlands).

### Statistics

**Figure 1:**
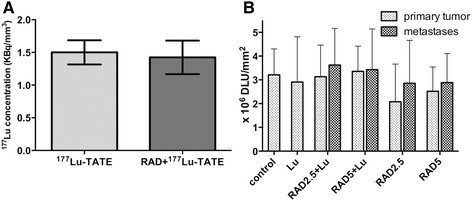
**Tumor concentration and quantification of sst**_**2**_**expression. (A)** Tumor concentration of 177Lu-DOTATATE in rats receiving 177Lu-DOTATATE-only (light grey bar) and groups receiving 177Lu-DOTATATE 4 days after RAD001 therapy was started (dark grey bar). **(B)** Quantification of sst_2_ expression in primary tumors and metastases based on *in vitro* autoradiography. ^177^Lu-TATE, ^177^Lu-DOTA^0^,Tyr^3^-octreotate; RAD, RAD001 2.5 or 5 mg/kg; DLU, digital light unit.

**Figure 2:**
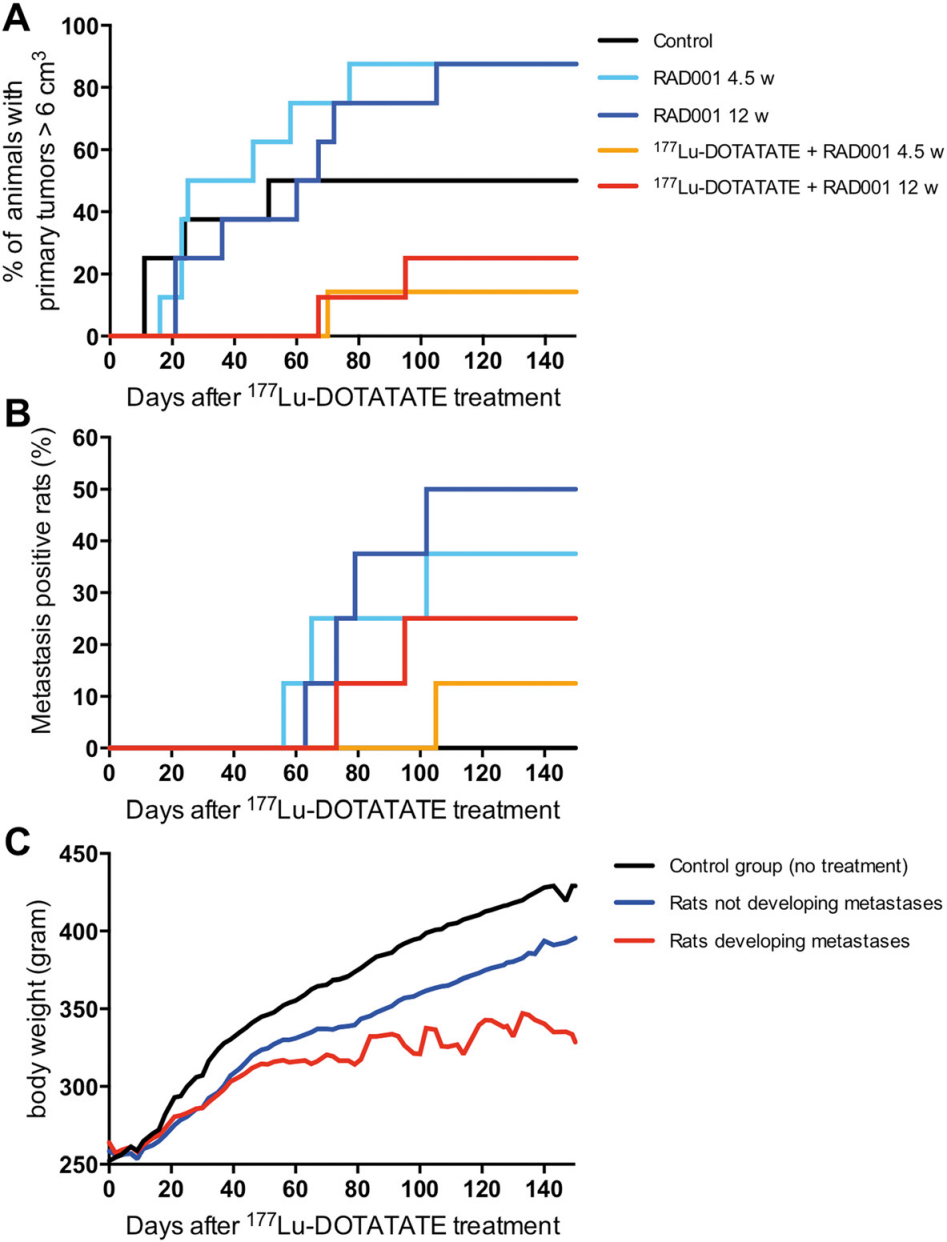
**Graphs showing percentage of rats having primary tumors and metastases, and mean body weight of animals. (A)** Percentage of (LEW/SsNHsd Lewis) rats with primary tumors reaching the maximum size of >6 cm3 that underwent surgery afterwards to remove the tumor. The control group received saline. RAD001 therapy started at day 4 (5 mg/kg administered twice weekly). ^177^Lu-DOTATATE (125 MBq) was administered at day 1. **(B)** Percentage of (LEW/SsNHsd Lewis) rats developing metastases in each group. **(C)** Mean body weight of animals in the control group (black line) and of the rats treated with either RAD001 or a combination of RAD001 and ^177^Lu-DOTATATE that did not develop metastases (blue line) versus the body weight of rats treated with RAD001 or a combination of RAD001 and ^177^Lu-DOTATATE that developed metastases (red line).

Prism software version 5.0 (Graph Pad) was used to analyze tumor growth and determine statistical significance between groups. An unpaired *T* test was used for statistical analysis of tumor uptake (Figure 1). Results are given as mean ± SD. A log rank test was performed for curve comparison in Figure 2A,B. Body weight data in Figure 2C are expressed as mean values.

### Experimental design

An overview of the different research questions (A to E) and treatment groups in all studies (1 to 3) is given in Table 1 and described below.

**Table 1:** Overview of the research questions and setup of the subsequent studies

**Research question (rat strain)**	**Study**	**Groups**	**Number**	^**177**^**Lu-TATE (MBq)**	**RAD001 (dose, period)**
(A) Potential synergistic effect of RAD001 in combination with 177Lu-TATE (LEW/HanHsd)	1	Control	7	-	Placebo
		RAD only	6	-	5.0 mg/kg, 4.5 w
		^177^Lu-TATE low dose	7	125	Placebo
		^177^Lu-TATE high dose	7	275	Placebo
		RAD + ^177^Lu-TATE low dose	7	125	5.0 mg/kg, 4.5 w
		RAD + ^177^Lu-TATE high dose	7	275	5.0 mg/kg, 4.5 w
(B) Prolonged follow-up of potential development of distant metastasis (LEW/HanHsd)	2	Control	8	-	Placebo
		Low-dose RAD	8	-	2.5 mg/kg, 4.5 w
		High-dose RAD	8	-	5.0 mg/kg, 4.5 w
		^177^Lu-TATE	7	125	Placebo
		^177^Lu-TATE + low-dose RAD	8	125	2.5 mg/kg, 4.5 w
		^177^Lu-TATE + high-dose RAD	7	125	5.0 mg/kg, 4.5 w
(C) Influence of RAD001 on tumor uptake of 177Lu-DOTATATE (LEW/HanHsd)	1 + 2	^177^Lu-TATE	21	125 or 275	Placebo
		RAD + ^177^Lu-TATE	29	125 or 275	2.5 mg/kg or 5.0 mg/kg, 4.5 w
(D) Effects of prolonged RAD001 treatment (LEW/SsNHsd)	3	Control	8	-	Placebo
		RAD	8	-	5.0 mg/kg, 4.5 w
		^177^Lu-TATE + RAD	8	125	5.0 mg/kg, 4.5 w
		RAD prolonged treatment	8	-	5.0 mg/kg, 12 w
		^177^Lu-TATE + RAD prolonged treatment	8	125	5.0 mg/kg, 12 w
(E) Effects of PRRT on growth of metastases (LEW/SsNHsd)	3	High-dose ^177^Lu-TATE after diagnosis of metastases	7	400	-

^177^Lu-TATE, ^177^Lu-DOTA^0^,Tyr^3^-octreotate; RAD, RAD001; PRRT, peptide receptor radionuclide therapy; w, weeks.

#### (A) Potential synergistic effect of RAD001 in combination with ^177^Lu-DOTATATE

To study whether RAD001 has an additional anti-tumor effect on ^177^Lu-DOTATATE, six different study groups were included with six to seven rats per group. Besides the control group and the RAD001-only therapy groups (5 mg/kg twice a week for 4.5 weeks), two groups received ^177^Lu-DOTATATE as a single therapy in different doses (125 or 275 MBq), and two groups received the same doses of ^177^Lu-DOTATATE combined with RAD001 treatment (5 mg/kg twice a week for 4.5 weeks starting 3 days prior to PRRT) [11]. Rats were euthanized when tumor size exceeded 4 cm^3^.

#### (B) Prolonged follow-up of potential development of distant metastasis in all groups after surgical resection of the primary tumor

Six additional groups of rats were included in the next study with seven to eight rats in each group. To enable prolonged follow-up, in this experiment, primary tumors were surgically removed when >4 cm^3^. This enabled longer follow-up to study the development of metastases in relation to the combination of ^177^Lu-DOTATATE and RAD001 or RAD001-only. RAD001 (2.5 or 5.0 mg/kg) was administered twice weekly for 4.5 weeks, either alone or 3 days prior to 125 MBq ^177^Lu-DOTATATE.

#### (C) Influence of RAD001 on tumor uptake of ^177^Lu-DOTATATE

^177^Lu-DOTATATE uptake in tumors was quantified based on SPECT/CT scans acquired 48 h after administration of ^177^Lu-DOTATATE in experiments 1 and 2 to examine if previous RAD001 treatment results in reduced ^177^Lu retention in CA20948 tumors.

#### (D) Effects of prolonged RAD001 treatment

Five groups of eight rats were included. RAD001 (5 mg/kg) therapy was started 4 days after 125 MBq ^177^Lu-DOTATATE. RAD001 was administered twice a week and continued for either 4.5 or 12 weeks. Surgical resection was performed if tumors reached a volume of 6 cm^3^.

#### (E) Effects of PRRT re-treatment on metastases

If rats in experiment 3 showed lethargy or a >10% body weight loss, SPECT/CT was performed using ^111^In-DTPA-octreotide. If metastases could be discriminated, 400 MBq/10.8 μg ^177^Lu-DOTATATE PRRT was given. Twenty-four hours after ^177^Lu-DOTATATE injections, SPECT/CT was performed to image ^177^Lu-DOTATATE uptake in metastases. Therapeutic effect was monitored by follow-up of body weight and ^111^In-DTPA-octreotide SPECT/CT when ongoing decrease in body weight was registered.

## Results

### (A) Potential synergistic effect of RAD001 in combination with ^177^Lu-DOTATATE

In experiment 1, treatment with RAD001-only did not result in any complete or partial anti-tumor responses, defined as follows: partial response (PR), which is >50% reduction of tumor volume, but no complete response (CR), which is 100% reduction of tumor volume (Table 2). Groups treated with ^177^Lu-DOTATATE-only showed 57% CR after 125 MBq ^177^Lu-DOTATATE and 71% after 275 MBq ^177^Lu-DOTATATE. Combination of ^177^Lu-DOTATATE and RAD001, however, resulted in only 29% CR after 125 MBq ^177^Lu-DOTATATE + RAD001 and 14% after 275 MBq ^177^Lu-DOTATATE + RAD001. So, in contrast to our hypothesis, no additive effect with regard to tumor response could be achieved by combining RAD001 and ^177^Lu-DOTATATE. Moreover, unexpectedly, all rats treated with the combination of RAD001 and ^177^Lu-DOTATATE and not showing CR eventually developed metastases as was reported earlier [11].

**Table 2:** Overview of tumor responses

**Experiment**	**Group**	**CR (%)**	**PR (%)**	**Rats with metastases (%)**	***n***
1	Control^a^	0	0	0	7
	RAD 5 mg/kg^a^	0	0	0	6
	^177^Lu-TATE 125 MBq	57	29	0	7
	^177^Lu-TATE 278 MBq	71	29	0	7
	RAD 5 mg/kg + ^177^Lu-TATE 125 MBq	29	57	71	7
	RAD 5 mg/kg + ^177^Lu-TATE 278 MBq	14	57	86	7
2	Control^b^	0	0	0	8
	RAD 5.0 mg/kg^b^	0	13	75	8
	RAD 2.5 mg/kg^b^	13	0	63	8
	^177^Lu-TATE 125 MBq^b^	43	57	0	7
	^177^Lu-TATE 125 MBq + RAD 5.0 mg/kg^b^	0	63	88	8
	^177^Lu-TATE 125 MBq + RAD 2.5 mg/kg^b^	14	43	86	7
3	Control^b^	50	0	0	8
	RAD 4.5 weeks^b^	12.5	12.5	37.5	8
	RAD 12 weeks^b^	12.5	25	50	8
	^177^Lu-TATE 125 MBq + RAD 4.5 weeks^b^	87.5	12.5	12.5	8
	^177^Lu-TATE 125 MBq + RAD 12 weeks^b^	75	25	25	8

^a^Rats did not survive until 42 to 146 days post start of treatment (p.t.), the time frame in which metastases became apparent in the other groups, because rats had to be euthanized when primary tumor size was >4 to 6 cm^3^. ^b^Primary tumors were surgically removed when tumor size was >4 to 6 cm^3^. CR, complete response (100% reduction of tumor size); PR, partial response (>50% reduction of tumor volume but no CR); *n*, number of animals/group; ^177^Lu-TATE, ^177^Lu-DOTA^0^, Tyr^3^-octreotate; RAD, RAD001.

### (B) Prolonged follow-up of potential development of distant metastasis in all groups after surgical resection of the primary tumor

Following prolonged monitoring after primary tumor removal, the majority (77%) of rats treated with RAD001 (either 5.0 or 2.5 mg/kg) developed metastases that resulted in mean body weight loss at around 43 days after start of treatment. For these two doses, no dose dependence of RAD001 was found (Table 2).

### (C) Influence of RAD001 on tumor uptake of ^177^Lu-DOTATATE

The ^177^Lu tumor uptake in ^177^Lu-DOTATATE-only-treated rats was 1.51 ± 0.07 kBq/mm^3^, while this was 1.42 ± 0.07 kBq/mm^3^ in rats pretreated with RAD001; no significant different values were found between the groups (*p* = 0.50, Figure 1A).

### (D) Effects of prolonged RAD001 treatment

In contrast to previous experiments, 50% of the rats in the control group showed a CR. The rats from the combination groups (^177^Lu-DOTATATE + RAD001 for 4.5 weeks and ^177^Lu-DOTATATE + RAD001 for 12 weeks) showed a CR in 87.5% and 75% (not significantly different; *p* = 0.63) of the animals, respectively, in comparison to only 12.5% of rats in both RAD001-only therapy groups. Within these two RAD001-only groups, there was no significant difference regarding both the number of animals that needed surgery as well as the time until surgery.

With regard to development of metastases, at day 150 post start of treatment (p.t.), no metastases were detected in untreated rats (Figure 2B). Also, the time of appearance of the metastases in the combination group was later: 61 d p.t. vs. 91 p.t., respectively.

Monitoring the body weight of rats revealed the effects of treatment and the development of metastases. Rats in the control group showed a normal gain in body weight over time (Figure 2C). Rats treated with ^177^Lu-DOTATATE + RAD001 or RAD001-only not developing metastases also showed gain in body weight over time, although at a slower rate. However, the mean body weight of rats developing metastases stabilized as a result of their poor condition.

### (E) Effects of PRRT on growth of metastases

**Figure 3:**
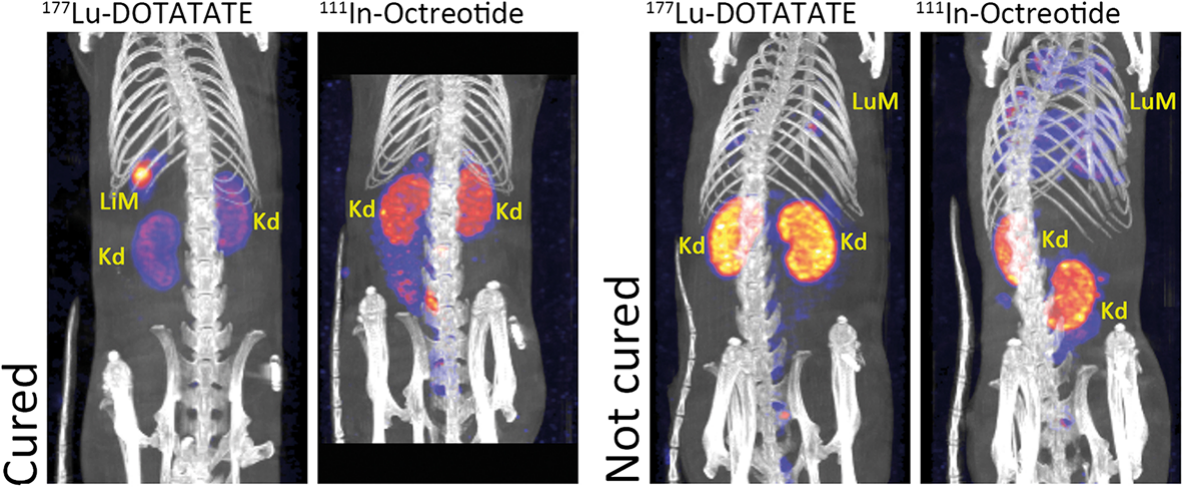
**Two representative sets of SPECT/CT scans of rats before and after re-treatment with**^**177**^**Lu-DOTATATE.** The two images at the left represent a rat with liver metastasis; the lesion was not detectable anymore on the scan with ^111^In-DTPA-octreotide made 8 days after ^177^Lu-DOTATATE PRRT. The two images at the right represent a rat treated for lung metastases. On the right, scan after injection of ^111^In-DTPA-octreotide was made before euthanasia of the rat because of ongoing weight loss. LuM, lung metastasis; LiM, liver metastases; Kd, kidney.

Although SPECT/CT confirmed significant uptake of ^177^Lu-DOTATATE in metastases (Figure 3), in only two of the seven rats, there was CR after re-treatment with high-dose ^177^Lu-DOTATATE. The average survival time of the non-responsive rats after detection and treatment of the metastases varied between 8 and 37 days, with an average of 27 days. One rat with CR recovered from a liver metastasis that was clearly visualized after administration of ^177^Lu-DOTATATE. Eight days later, there was no sign of this lesion as shown in the ^111^In-octreotide scan, which was confirmed after dissection. On the other hand, a rat suffering from lung metastases did not respond to ^177^Lu-DOTATATE. Eight days after re-treatment, further loss in body weight was measured and the ^111^In-DTPA-octreotide SPECT/CT still showed extended lung metastases, also found at autopsy. Determination of sst_2_ density on CA20948 primary tumors and metastases using *in vitro* autoradiography revealed no significant differences (Figure 1B).

## Discussion

We previously reported on the disappointing results of the combination of mTOR inhibitor RAD001 with ^177^Lu-DOTATATE PRRT in the CA20948 rat tumor model. No hypothesized synergistic effect was found; the combination treatment appeared even less effective than ^177^Lu-DOTATATE-only. This observation cannot be explained by reduced ^177^Lu-DOTATATE uptake in the subcutaneous CA20948 tumors after RAD001 treatment, as we demonstrated here (Figure 1A).

Even more striking was the fact that the majority of RAD001-treated animals developed tumor metastasis to lymph nodes, liver, and/or lung. RAD001 initially has been introduced as an immunosuppressant to protect patients from rejecting allografts after organ transplantation [22]. In 2005, Law et al. [23] reviewed the immunosuppressive effects of RAD001 in relation to its anti-tumor effects and discussed immune suppression by RAD001 to be tumor growth accentuating. Therefore, the application of RAD001 as an anti-tumor agent should be monitored carefully in the clinic, but to the best of our knowledge as yet, no tumor accentuating effects in patients have been reported. Recently, after the publication of our priority report on our first findings, RAD001 has received FDA approval for the treatment of advanced NETs and is commonly used in clinical practice nowadays. Although we must consider the fact that RAD001 is used in patients with already advanced (metastasized) disease, so far, no reports on accelerated metastasis in patients related to RAD001 treatment have been published. We earlier hypothesized multiple reasons for the occurrence of metastases: the twice-weekly dose regimen instead of daily dosing as is applied in clinical therapy, effects of RAD001 on the immune system and/or tumor microenvironment, or the discontinuation of RAD001 treatment at 4.5 weeks after start of treatment. In the current studies, we compared 4.5 weeks of RAD001 treatment with 12 weeks of twice-weekly RAD001 treatment. This prolonged RAD001 treatment (with or without ^177^Lu-DOTATATE therapy) did not reduce the number of rats developing metastases, and in addition, no delay in the occurrence of metastasis was seen. In fact, comparison of the 4.5-week RAD001 groups versus 12-week RAD001 groups showed a higher percentage of rats developing metastasis in the 12-week RAD001 groups than in the 4.5-week RAD001 groups, namely, 38% vs. 25% (*p* = 0.45). The average time until detection of metastases was also not significantly different between groups receiving RAD001 for 4.5 weeks (82 days p.t.) versus 12 weeks (78 day p.t.). Moreover, in 67% of the rats developing metastases in the 12-week treatment groups, metastases were diagnosed while rats were still receiving RAD001. Therefore, it is unlikely that the occurrence of metastases is due to cessation of RAD001 administration. Potentially, the twice-weekly administration could lead to a repetitive on-off effect on the mTOR pathway with repetitive upregulation/rebound effects of the mTOR pathway with varying plasma concentration levels of RAD001 in a twice-weekly dose regimen [24]. In future experiments, a daily RAD001 dose regimen will have to be compared to the twice-weekly dose regimen as was used in the current studies.

Compared to a treatment with RAD001-only, less animals receiving a combination of ^177^Lu-DOTATATE and RAD001 developed metastases, and mean time to diagnosis for those metastases was 30 days later compared to the rats receiving RAD001-only. Results from the combination therapy groups with ^177^Lu-DOTATATE administered 4 days before RAD001 therapy suggested that ^177^Lu-DOTATATE administered prior to RAD001 therapy reduced both incidence and time of onset of metastases in comparison to the reverse order combination. When ^177^Lu-DOTATATE was administered 4 days after RAD001 therapy, however, there was no reduction in the percentage of rats developing metastases in the combination therapy groups, indicating that rats with (some) tumor reduction already induced by PRRT were less likely to develop metastases than rats not treated with PRRT. This is in agreement with the fact that animals that showed CRs after PRRT or PRRT plus RAD001 did not develop metastases during follow-up.

In studies 1 and 2, we used a syngeneic tumor model in rats with an uncompromised immune system. A significant role for T lymphocytes in the immune response to tumors after or during ionizing radiation therapy has been described, the latter resulting in upregulation of tumor-specific antigens [25-29]. As immune suppression by RAD001 has been proven to be mainly due to suppression of T lymphocyte activation and proliferation [30,31], in our opinion, immune suppression by RAD001 is a likely explanation for reduced tumor response to PRRT in combination with RAD001 as observed in our study. In studies 1 and 2, LEW/HanHsd rats were used, whereas in study 3, the LEW/SsNHsd substrain was used, providing the opportunity to test the hypothesis mentioned above. In this substrain, the immune system is more active compared to the HanHsd strain and shows an enhanced (auto)-immune response [13-17]. In these rats, 50% of tumors in the control group went into spontaneous regression after reaching an average tumor volume of ≈ 3 cm^3^, probably due to an immune response against the growing tumor. In such rats treated with RAD001 (4.5 or 12 weeks), only 12.5% of the tumors went into regression. In a mouse model on rejection of an allogeneic subcutaneous tumor as created by Hammond-McKribben et al. [22], another mTOR inhibitor, the rapamycin derivate SDZ RAD, was used to prevent rejection of allogeneic tumors. So, immune suppression by RAD001 might have caused the reduced tumor response in our study with ^177^Lu-DOTATATE administered after RAD001 as well.

Contrary to our results, a combination of ionizing radiation (IR) with rapamycin has been proven to be more effective than IR-only in preclinical studies [10,32,33]. These studies however have been performed in xenograft models using immunodeficient mice lacking T cells.

As discussed in our priority report, reduced cell proliferation rate caused by a G1 arrest could also be an explanation for the reduced tumor response to ^177^Lu-DOTATATE in rats treated with the combination of RAD001 and ^177^Lu-DOTATATE. RAD001 treatment has been shown to cause a G1 arrest, as mTOR is being linked to phosphatidylinositol 3-kinase (PI3K) pathways [34]. Within 24 h after RAD001 administration, a significant increase of cells in G1 phase has been described [35,36]. As cells with a long cycling time, including NET cells, have a peak of radioresistance during early G1 phase [37], tumor cells may have been less sensitive to ^177^Lu-DOTATATE when administered 4 days after the start of RAD001 treatment. Since clinical trials combining RAD001 and PRRT are being performed [38], in our opinion, the decreased anti-tumor response in our study when RAD001 was administered prior to ^177^Lu-TATE is rather relevant. Also, in a clinical situation, the combination of both therapies might be less effective compared to just PRRT.

For re-treatment of the rats with metastases, we used a dose of 400 MBq ^177^Lu-DOTATATE, which is remarkably higher compared to the initial 125 or 275 MBq treatment doses. Still, only two out of seven rats with metastases were cured, suggesting metastases in our model to be more resistant to PRRT compared to the primary tumor. As it is quite complex to study responses of metastases in a preclinical model, there have not been many preclinical studies on therapies in metastatic models. However, to be able to get more solid information on sensitivity of metastases to PRRT in a preclinical model, certainly more studies in different models are necessary. The current CA20948 metastatic tumor model after RAD001 treatment could be a more realistic metastasis model for future experiments compared to often-applied metastasis tumor models in which tumor cell suspensions are injected intravenously.

## Conclusions

Results described here confirmed and provided more information on development of metastasis after RAD001 treatment in our *in vivo* rat tumor model. The impaired tumor response to the combination of RAD001 and ^177^Lu-DOTATATE in comparison with that after ^177^Lu-DOTATATE-only could not be attributed to a reduced tumor uptake of ^177^Lu-DOTATATE in rats after RAD001 treatment. Moreover, the occurrence of metastases could not be attributed to the sudden cessation of RAD001 treatment, as we observed treatment for 12 weeks did not result in a lower metastasis rate compared to treatment for 4.5 weeks. Immune suppression by RAD001 could be a good explanation for reduced tumor response after RAD001 as well as for development of metastasis. More studies in different tumor models are needed now to provide proof and give detailed information on the translational value of these findings to the clinic.

## Competing interest

The authors declare that they have no competing interests.

## Authors' contributions

SEP and SMB performed part of the animal experiments, participated in the design of the studies, and wrote the manuscript. SJK performed the animal experiments, SPECT/CT scanning, and data interpretation. LvdG performed the *in vitro* autoradiography (including quantification) and interpretation. HCG performed the quantification of SPECT data. MM and MdJ participated in its design and coordination and helped draft the manuscript. All authors read and approved the final manuscript.

## Authors' information

SMB and SEP are the first authors.
